# When Mitochondria Falter, the Barrier Fails: Mechanisms of Inner Blood-Retinal Barrier (iBRB) Injury and Opportunities for Mitochondria-Targeted Repair

**DOI:** 10.3390/ijms262411984

**Published:** 2025-12-12

**Authors:** Ziyi Chen, Qianzi Jin, Jiajun Li, Keran Li

**Affiliations:** 1 Department of Ophthalmology, The Affiliated Eye Hospital of Nanjing Medical University, Nanjing 210029, China; 2 The Fourth School of Clinical Medicine, Nanjing Medical University, Nanjing 211166, China

**Keywords:** inner blood-retinal barrier, mitochondrial dysfunction, oxidative stress, mitophagy, diabetic retinopathy, retinal vein occlusion, mitochondrial therapy, mitochondrial plasticity

## Abstract

As the central hub of retinal metabolism, mitochondria are vital for sustaining the integrity of the inner blood-retinal barrier (iBRB), which is fundamental to retinal homeostasis. Mitochondrial dysfunction accelerates severe iBRB disruption, a process which is increasingly implicated in a cascade of mitochondrial pathologies including mitochondrial DNA destabilization, oxidative stress, calcium homeostasis disruption, mitochondrial autophagy deficiency, and dysregulated dynamic regulation. This review establishes the iBRB as a crossroads for metabolic, redox, and inflammatory signaling. By analyzing evidence from diabetic retinopathy and retinal vein occlusion models, we clarify how mitochondrial decline translates local energy deficiency into chronic barrier dysfunction. We posit that restoring mitochondrial function is indispensable for vascular resilience and regeneration, a conclusion drawn from integrating molecular, cellular, and translational findings. To advance mitochondrial discoveries into clinical practice, subsequent studies must prioritize achieving spatiotemporally controlled, cell-type-specific interventions with robust in vivo efficacy, thereby successfully translating mitochondrial science into clinical vascular medicine.

## 1. Introduction

**Figure 1:**
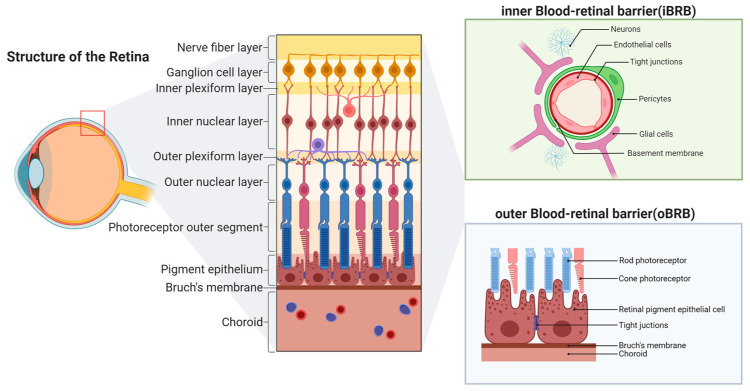
Structural organization of the inner blood-retinal barrier (iBRB). The blood-retinal barrier (BRB) comprises two components: the inner (iBRB) and outer (oBRB) barriers. The iBRB is formed primarily by retinal vascular endothelial cells and their tight junctions. Together with the endothelial basement membrane, pericytes, glial cells, and neurons, these components keep the inner barrier structurally and functionally intact. The oBRB, shown for anatomical context, is formed by the retinal pigment epithelium and Bruch’s membrane overlying the choroid. The figure was created in BioRender (Chen, Z. (2025) https://BioRender.com/8mnbjpf).

The blood-retinal barrier (BRB) plays a crucial role in maintaining retinal homeostasis, regulating the exchange of substances between the retina and the bloodstream, and preserving the stability of the internal environment [1,2]. The BRB can be divided into the inner blood-retinal barrier (iBRB) and the outer blood-retinal barrier (oBRB) [3]. The iBRB comprises retinal vascular endothelial cells and their tight junctions. The structural integrity of the inner barrier is collectively maintained by the endothelial cell basement membrane, pericytes, glial cells, neurons and other components [4], with the permeability of macromolecules and cells being collectively restricted (Figure 1). This barrier is vital for protecting the retina from harmful substances and maintaining the normal function of retinal neurons and blood vessels [5]. Disruption of the iBRB is an early and decisive event in blinding retinal diseases such as diabetic retinopathy (DR) and retinal vein occlusion (RVO), triggering vascular leakage, inflammation and neurodegeneration [6,7,8,9,10,11].

The retina is one of the most metabolically active tissues in the human body. It requires continuous, high-throughput ATP production to sustain phototransduction and synaptic activity. This exceptional energy demand makes retinal cells, particularly photoreceptors, Müller glia and vascular endothelial cells, highly dependent on mitochondrial function. As the central hub of cellular bioenergetics, mitochondria generate ATP through oxidative phosphorylation (OXPHOS) while also coordinating redox balance, calcium homeostasis and apoptotic signaling. These processes are all indispensable for retinal vitality and iBRB integrity.

The iBRB and its supporting cells are highly susceptible to mitochondrial stress. Consequently, mitochondrial dysfunction, manifested as disturbances to energy metabolism, excessive ROS generation, impaired mitophagy and disrupted mitochondrial dynamics, directly compromises barrier integrity. This leads to endothelial and pericyte injury, vascular leakage and progressive structural collapse [12].

Although there is a growing amount of evidence establishing a strong link between mitochondrial dysfunction and blinding retinal diseases, the specific ways in which different types of mitochondrial impairment, such as mtDNA mutations, excessive ROS generation, defective mitophagy and disturbed mitochondrial dynamics, affect the iBRB and its supporting cells are not well understood. In particular, the molecular interplay connecting mitochondrial failure to barrier disintegration has not been systematically delineated across different retinal cell types. The aim of this review is to integrate current mechanistic insights, clarify how mitochondrial dysfunction disrupts the structural and metabolic integrity of the iBRB and identify potential therapeutic avenues that target mitochondrial regulation to preserve retinal vascular homeostasis.

Although the focus of this review is the inner BRB, it is important to note that mitochondrial dysfunction also affects the retinal pigment epithelium (RPE) that forms the outer barrier. The metabolic demands, stress responses, and mitochondrial regulatory programs of the oBRB differ markedly from those of iBRB endothelial cells and pericytes, and thus are referenced here only briefly to maintain the mechanistic focus on iBRB integrity.

## 2. Mitochondrial Dysfunction and Its Molecular Mechanisms

**Figure 2:**
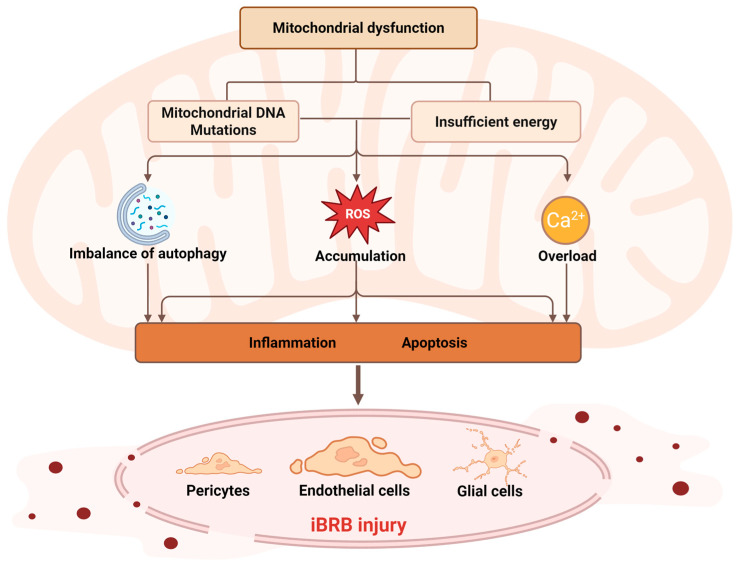
Mechanisms of iBRB injury induced by mitochondrial dysfunction. Pathogenic mtDNA mutations impair mitochondrial transcription and oxidative phosphorylation (OXPHOS), resulting in decreased ATP synthesis and metabolic insufficiency. The ensuing energy deficit triggers excessive mitochondrial reactive oxygen species (ROS) generation, dysregulated mitophagy, and Ca2+ overload. These interacting maladaptive processes amplify oxidative stress and inflammatory signaling, ultimately inducing apoptosis in endothelial cells and disrupting the structural and supportive functions of pericytes and glial cells. Together, these cell-specific injuries drive the progressive destabilization and breakdown of the inner blood-retinal barrier (iBRB). The figure was created in BioRender (Chen, Z. (2025) https://BioRender.com/8eswc2l).

Mitochondrial dysfunction has emerged as a key factor in iBRB injury, causing a range of bioenergetic, oxidative and structural disruptions in different types of cells. This dysfunction is rooted in mitochondrial DNA (mtDNA) mutations and transcriptional defects, which compromise OXPHOS and ATP production, precipitating a state of metabolic insufficiency. The resulting energy crisis intensifies mitochondrial ROS generation, shifting redox signaling into oxidative stress. In parallel, excess ROS destabilize mitochondrial quality control by impairing mitophagy, disrupt the delicate regulation of calcium homeostasis, and bias the equilibrium of mitochondrial dynamics toward fragmentation and loss of adaptive capacity (Figure 2). These maladaptive processes form a self-perpetuating loop that progressively entraps endothelial cells, pericytes, and glial cells in a feed-forward cycle of injury (Table 1). The following sections explore these pathways in detail, elucidating how their interactions converge on the progressive structural and functional breakdown of the iBRB.

### 2.1. Mitochondrial DNA Mutations

mtDNA, a small circular genome located in the mitochondrial matrix, encodes critical subunits of OXPHOS complexes as well as enzymes involved in energy metabolism, calcium signaling, and apoptosis regulation [6,13,14]. Unlike nuclear DNA, mtDNA replicates and transcribes independently, which provides a degree of autonomy but also makes it highly susceptible to mutations. Mutations in mtDNA—including point mutations and large deletions—lead to heteroplasmy, a state in which mutant and wild-type mtDNA coexist within the same cell [15,16]. An excessive ratio of mutant mtDNA significantly impairs the assembly and activity of OXPHOS complexes, reduces electron transport chain (ETC) efficiency, and escalates mitochondrial ROS production, thus initiating a cycle of oxidative stress and energetic failure [17,18].

Compromised ATP synthesis leads to dissipation of mitochondrial membrane potential (ΔΨm), collapse of the proton gradient, and inadequate energy production, ultimately compromising endothelial survival and barrier integrity [19,20]. Furthermore, damaged mtDNA may translocate into the cytoplasm, where it acts as a damage-associated molecular pattern (DAMP), activating innate immune pathways and amplifying local inflammation.

Within the iBRB, endothelial cells appear particularly susceptible to mtDNA insults. Mutations in genes encoding components of Complex I (e.g., *ND1, ND6*) and cytochrome b reduce endothelial cell proliferation and heighten apoptotic vulnerability, thereby exacerbating tight junction disruption and promoting iBRB destabilization [21,22,23,24,25,57,58,59]. Recent evidence further shows that even impaired mitochondrial transcription, such as from *POLRMT* (mitochondrial RNA polymerase) depletion, can independently trigger endothelial apoptosis, solidifying mtDNA as a key vulnerability node in iBRB pathology.

### 2.2. Cellular Energy Supply and Metabolic Disorders

Following the genetic and transcriptive insults, the most immediate consequence of mitochondrial dysfunction is a profound cellular energy crisis. Mitochondria synthesize the vast majority of cellular ATP via OXPHOS [31]. In this process, the ETC oxidizes NADH and FADH2 to generate the energy needed to drive ATP synthase. Mitochondrial dysfunction, instigated by mtDNA mutations and other factors, leads to a marked reduction in this ATP output, depriving iBRB cells of the energy required for their metabolic and survival pathways.

ATP scarcity impairs the activity of key enzymes such as ATP synthase and pyruvate dehydrogenase, further weakening OXPHOS. This deficit is exacerbated by other co-occurring pathologies: calcium overload, which collapses the membrane potential and suppresses ATP generation, and the overproduction of ROS, which inhibits transcription factors like TFAM and damages mtDNA, further reducing the expression of OXPHOS components. These interconnected disturbances create a self-reinforcing cycle of energy failure.

The resulting energy deficit is particularly devastating for the iBRB, which relies on a constant, high-energy supply to maintain its barrier function [26,32,50,60,61]. For endothelial cells, ATP deficiency impairs cytoskeletal dynamics, ion homeostasis, and vesicle trafficking, all of which are essential for maintaining tight junction integrity and barrier repair [52,62]. In pericytes, reduced ATP impairs contractility and paracrine signaling, destabilizing microvascular tone. Astrocytes and Müller glia also suffer, as mitochondrial dysfunction weakens their structural support and regulatory feedback on endothelial cells, culminating in barrier destabilization [33]. Beyond direct energetic failure, ATP deficiency compromises AMPK signaling, a crucial pathway that enhances cellular resistance to oxidative and inflammatory stress. Collectively, these widespread energetic deficits amplify cellular injury and accelerate iBRB breakdown.

### 2.3. ROS Generation and Oxidative Stress

Mitochondria are not only the center of cellular energy metabolism but also the primary site of reactive oxygen species (ROS) production. Within the ETC, complex I (NADH: ubiquinone oxidoreductase) and complex III (ubiquinol: cytochrome c oxidoreductase) are major contributors to ROS generation [35]. Under physiological conditions, low levels of ROS play regulatory roles in cell signaling, gene expression, and chromatin remodeling [63]. For instance, under hypoxia, mitochondria may increase ROS production as an adaptive signal to help cells cope with environmental stress [64]. However, high concentrations of ROS exhibit strong chemical reactivity, which can induce lipid peroxidation, protein modification, and enzyme inactivation, leading to widespread damage to cellular organelles [65]. MtDNA is particularly vulnerable to ROS due to its lack of histone protection and limited repair mechanisms, leading to downregulation of OXPHOS-related genes and forming a vicious cycle of “ROS accumulation–mtDNA damage–metabolic failure.” This vicious loop directly intersects with calcium overload, mitophagy imbalance, and mitochondrial fragmentation, creating a multifactorial stress network that progressively erodes iBRB integrity.

Within the iBRB, the maintenance of endothelial function relies heavily on redox balance [66,67]. Excessive mitochondrial ROS initiate a convergent attack that dismantles the iBRB by compromising the endothelial cells. ROS-mediated stress rapidly activates key inflammatory platforms, including the NLRP3 inflammasome and the JNK/p38 MAPK cascades, driving multiple forms of programmed cell death (PCD) in endothelial cells (including pyroptosis, parthanatos, and ferroptosis). Concurrently, ROS-mediated NF-κB activation promotes the release of pro-inflammatory factors and permeability enhancers such as IL-6 and VEGF. This inflammatory and apoptotic milieu directly results in the downregulation of essential tight junction proteins (ZO-1, Occludin, Claudin-5). Crucially, this structural compromise is amplified by enzymatic degradation, as ROS-driven signaling pathways promote the activation of matrix metalloproteinases (MMP-2/9), which directly cleave and degrade the extracellular domains of the junctional complexes. Moreover, ROS exacerbate the damage through organelle crosstalk, notably by amplifying stress via the IP3R–GRP75–VDAC1 axis [68], which links mitochondrial and endoplasmic reticulum stress to further exacerbate iBRB disruption.

The effects of oxidative stress are distinct and devastating in supporting cells. In pericytes, ROS disrupt cytoskeletal dynamics and inhibit contractile function by oxidizing actomyosin components and impairing ATP-dependent signaling, thereby weakening vascular tone regulation. Moreover, mitochondrial oxidative stress promotes pericyte apoptosis via Bax translocation and cytochrome c release, leading to a reduction in pericyte coverage—a key structural event in early vasodegeneration. In summary, oxidative stress should be regarded as a central hub linking mitochondrial dysfunction, inflammatory signaling, and vascular degeneration.

### 2.4. Regulation of Mitophagy

Mitophagy is a selective process by which cells remove damaged mitochondria, thus maintaining mitochondrial quality and preventing excessive oxidative stress and cell death. This process is primarily regulated by the PINK1/Parkin axis and outer membrane receptors (e.g., BNIP3, FUNDC1). When the mitochondrial membrane potential is lost, PINK1 accumulates on the outer membrane and recruits the ubiquitin ligase Parkin, which ubiquitinates mitochondrial membrane proteins and targets them for recognition by autophagosomes and subsequent degradation through the lysosomal pathway [39]. Under physiological stress, this pathway is typically protective, maintaining mitochondrial quality and preventing excessive oxidative stress. Under pathological conditions, however, mitophagy becomes imbalanced and cell-type-specific, transforming its protective role into a pathogenic driver of iBRB injury.

In endothelial cells, mitophagy dysregulation is predominantly characterized by insufficient clearance due to defective PINK1/Parkin signaling and flux blockade. PINK1/Parkin failure impairs the recognition of damaged mitochondria, while parallel alterations in AMPK–mTOR signaling further contribute to this defect: persistent activation of mTORC1 blocks autophagosome maturation, thereby producing functionally defective mitophagy [40,69,70]. As damaged mitochondria accumulate, persistent mitochondrial ROS elevation and mtDNA instability fuel chronic inflammation. This involves the activation of inflammasomes and downstream NF-κB signaling, which promotes the release of pro-inflammatory cytokines, enhances apoptosis, and directly leads to structural failure [36,71,72]. The accumulated damage causes the downregulation of essential tight junction proteins and disrupts cytoskeletal organization, collectively resulting in endothelial barrier leakage [37,41]. Furthermore, persistent deficiency facilitates mtDNA leakage into the cytoplasm, sustaining sterile inflammation and amplifying endothelial dysfunction indirectly through paracrine signaling [55]. Experimental evidence shows that pharmacological restoration of mitophagy suppresses cytokine release and preserves tight junction integrity, underscoring the causal link between impaired clearance and barrier failure.

In contrast, the pathological pattern in pericytes is often distinct and paradoxical: stress conditions frequently induce overactivation rather than deficiency. For example, TXNIP upregulation, particularly in the diabetic milieu, drives excessive mitophagy that progressively depletes mitochondrial content. This vulnerability is exacerbated by Drp1-mediated mitochondrial fragmentation, which increases the proportion of mitochondria targeted for clearance. The result is a profound bioenergetic collapse, heightened oxidative stress, and rapid apoptosis of the pericytes. Loss of pericyte coverage reduces structural support for endothelial cells, destabilizes capillaries, and accelerates vasodegeneration, a hallmark of early DR. Animal models confirm that suppressing TXNIP or restraining excessive mitophagy protects pericyte survival and delays vascular regression [56,73]. This conceptual tension—where insufficient mitophagy harms endothelial cells while excessive mitophagy destroys pericytes—reveals that mitophagy functions not as a uniform housekeeping process but as a finely balanced, cell-type-specific regulator. Therefore, future therapeutic strategies must aim for dynamic tuning of mitophagic activity, rather than uniform enhancement, to restore iBRB homeostasis.

### 2.5. Regulation and Dysregulation of Calcium Homeostasis

Mitochondria serve as central regulators of intracellular calcium ion (Ca^2+^) homeostasis, employing specialized transport systems to dynamically modulate ionic fluxes and thereby couple Ca^2+^ signaling to energy metabolism and cell survival. The mitochondrial calcium uniporter (MCU) mediates rapid Ca^2+^ uptake, whereas the sodium/calcium/lithium exchanger (NCLX) and the calcium/hydrogen exchanger (CHE) support Ca^2+^ extrusion, together ensuring the spatiotemporal precision of mitochondrial Ca^2+^ signaling that safeguards against cytotoxic overload [42,43].

Under physiological conditions, moderate mitochondrial Ca^2+^ concentrations (<40 nmol/mg) activate key metabolic enzymes such as pyruvate dehydrogenase and α-ketoglutarate dehydrogenase, thereby promoting the tricarboxylic acid (TCA) cycle and ATP synthesis. However, during Ca^2+^ overload (>500 nmol/mg), the mitochondrial membrane potential collapses, OXPHOS becomes uncoupled, and ROS are transiently overproduced. These changes favor the aberrant opening of the mitochondrial permeability transition pore (mPTP), initiating mitochondrial dysfunction and cell death [44,45]. Thus, Ca^2+^ acts as both a bioenergetic regulator and a potential trigger for mitochondrial catastrophe, with its impact dictated by the intensity and duration of the signal.

Spatiotemporal regulation of Ca^2+^ in the retinal microvasculature influences iBRB homeostasis [46]. TRPV4-mediated Ca^2+^ influx can activate IP3 receptors on the endoplasmic reticulum (ER), generating rhythmic Ca^2+^ oscillations. Mitochondria positioned at ER–plasma membrane contact sites rapidly buffer these oscillations, stabilizing endothelial signaling and supporting tight junction integrity [34]. Under hyperglycemic stress, however, this homeostatic coordination collapses. Excessive activation of TRPV4 channels promotes sustained cytosolic Ca^2+^ influx and excessive MCU-mediated mitochondrial uptake, leading to Ca^2+^ overload in the matrix. The resulting suppression of ATP synthase activity, loss of mitochondrial membrane potential, and excessive ROS generation culminate in mitochondrial fragmentation and energy failure [38,74,75]. These changes compromise tight junction architecture—most notably ZO-1 degradation—and weaken the iBRB.

Moreover, mitochondrial Ca^2+^ overload serves as a secondary amplifier of inflammatory signaling. Abnormally enhanced Ca^2+^ signaling exerts dual deleterious effects. On one hand, elevated cytosolic Ca^2+^ activates Ca^2+^-dependent proteases such as calpain, which promote the assembly of the NLRP3 inflammasome and the maturation and release of downstream cytokines, directly contributing to endothelial injury. On the other hand, excessive accumulation of Ca^2+^ within the mitochondrial matrix activates the Drp1 pathway, leading to excessive mitochondrial fission and network fragmentation. This process disrupts mitochondrial autophagy and dynamic homeostasis, and further stimulates NF-κB and NLRP3 signaling cascades, thereby sustaining a proinflammatory response. As a result, key vascular permeability mediators—including IL-1β, TNF-α, and VEGF—remain persistently upregulated, aggravating endothelial barrier disruption and reinforcing the pathological cycle of mitochondrial dysfunction and calcium dyshomeostasis [47].

Importantly, these Ca^2+^-driven pathological cascades are not restricted to endothelial cells. In pericytes, dysregulated Ca^2+^ signaling impairs contractility and disrupts metabolic coupling with adjacent endothelial cells, thereby compromising vascular tone and barrier stability. Meanwhile, activated glial and neuronal cells within the inflammatory microenvironment release additional cytokines and vasoactive molecules, forming a self-amplifying feedback loop that further exacerbates microvascular injury.

Collectively, Ca^2+^ homeostatic imbalance represents a central pathogenic nexus in iBRB dysfunction, integrating mitochondrial impairment, redox imbalance, and inflammatory signaling across all cellular constituents of the iBRB.

### 2.6. Mitochondrial Dynamics Balance (Fusion and Fission)

Mitochondrial quality control is maintained by a dynamic interplay between fusion, fission, mitophagy, and biogenesis. Under physiological conditions, these processes constitute a tightly regulated network ensuring mitochondrial health. Fusion, mediated by mitofusins (MFN1/MFN2) and OPA1, allows for the exchange of contents and functional complementation between mitochondria. Conversely, fission, driven by Drp1 and its receptors like Fis1, segregates damaged mitochondrial segments for targeted removal via mitophagy. This cycle of renewal is completed by biogenesis, a process orchestrated by master regulators such as PGC-1α and nuclear respiratory factors (NRF1/NRF2), which replenishes the mitochondrial pool [76,77].

Under pathological conditions, abnormal expression or activity of mitochondrial dynamics regulators disrupts the balance between fusion and fission [48]. This imbalance is not an isolated event but is tightly intertwined with Ca^2+^ dyshomeostasis and defective mitophagy, together forming a self-reinforcing network of mitochondrial functional decline. In the retinal vasculature, hyperglycemia and oxidative stress excessively activate Drp1/FIS1, shifting the dynamic equilibrium toward excessive fission and mitochondrial fragmentation. Meanwhile, the expression of MFN2 and OPA1 is frequently downregulated, limiting the mitochondrial network’s capacity for repair, metabolic buffering, and stress adaptation. The resulting structural disruption further impairs OXPHOS, promotes cytochrome c release, and induces PCD in barrier-forming cells [19].

These alterations are most pronounced in retinal microvascular endothelial cells, where fragmented mitochondria are closely associated with tight-junction disassembly and increased barrier permeability. Similar patterns are also observed in pericytes, characterized by reduced contractility and compromised perivascular stability. In addition, suppression of mitochondrial biogenesis, largely associated with downregulation of PGC-1α, often exacerbates cellular dysfunction by diminishing the ability of cells to restore healthy mitochondrial populations and maintain energetic homeostasis.

Taken together, these findings establish mitochondrial dynamics as both a sensor and an effector of iBRB pathology. However, this central role also presents a significant therapeutic challenge. Interventions are not straightforward; for instance, inhibiting Drp1 to prevent fragmentation may reduce apoptosis but could inadvertently impair essential processes like mitophagy. Therefore, the future of therapeutic strategies lies not in unidirectional manipulation but in the restoration of mitochondrial plasticity—re-establishing the cell’s ability to adapt its mitochondrial network to meet physiological demands—which is the true therapeutic target.

## 3. Mechanisms of iBRB Damage Induced by Mitochondrial Dysfunction in Retinal Diseases

The integrity of the iBRB is essential for maintaining retinal structure and function. In clinical scenarios, iBRB damage is closely associated with retinal diseases such as DR and RVO [78,79]. Having delineated the core mitochondrial mechanisms underlying iBRB dysfunction, we next discuss their disease-specific manifestations in DR and RVO.

### 3.1. Diabetic Retinopathy (DR)

In DR, chronic hyperglycemia acts not merely as a generic stressor, but as a complex metabolic disruptor that activates specific non-canonical pathways, accelerating the generalized mitochondrial dysfunction mechanisms. This environment imposes unique, persistent molecular pressures, resulting in differential pathological outcomes across the iBRB’s cellular constituents [80]. The resulting pathology moves beyond simple bioenergetic failure, incorporating cell-specific quality control paradoxes and the long-term programming of inflammation known as metabolic memory.

The initial insult in DR involves the overloading of multiple glucose metabolic pathways. Hyperglycemia-induced flux through the Hexosamine Biosynthesis Pathway (HBP) leads to the accumulation of UDP-GlcNAc and subsequent global protein O-GlcNAcylation [81]. This post-translational modification is a key, DR-specific mechanism that directly targets and dysregulates mitochondrial homeostasis by increasing the activity of the fission mediator Drp1 and inhibiting key OXPHOS components, thereby predisposing the network to fragmentation and oxidative stress independent of standard ETC substrate overflow [82,83]. Concurrently, the activation of the Polyol Pathway depletes cytosolic NADPH pools, severely compromising the cell’s capacity to regenerate GSH and neutralize ROS, making the retina highly susceptible to unchecked oxidative damage. Furthermore, the formation of Advanced Glycation End-products, AGEs, and their binding to the RAGE receptor amplifies oxidative stress via NADPH oxidase NOX activation, establishing a sustained, compounded source of ROS that synergistically destabilizes mitochondrial integrity [84]. This metabolic milieu precipitates a severe energy crisis, characterized by a persistent drop in ATP synthesis that compromises the cytoskeletal dynamics of endothelial cells and the contractility of pericytes, leading to immediate barrier compromise.

Crucially, the regulation of mitochondrial quality control becomes distinctly cell-type specific in DR, reflecting a pathological paradox. In endothelial cells, the system is characterized by insufficient and functionally defective mitophagy. While mitochondrial dynamics are skewed toward excessive fission Drp1 upregulation), the resulting small, fragmented organelles often possess only partially depolarized membranes, enabling them to evade complete recognition and clearance by the PINK1/Parkin axis [85,86,87,88,89]. The accumulation of these dysfunctional fragments sustains high levels of mitochondrial ROS (mtROS) and mtDNA instability, fueling chronic inflammation and accelerating endothelial apoptosis. In stark contrast, pericytes exhibit pathological over-activation of mitophagy, predominantly driven by the high-glucose-induced upregulation of Thioredoxin-Interacting Protein TXNIP. TXNIP promotes uncontrolled clearance of mitochondrial mass, leading to a profound bioenergetic collapse and rapid pericyte loss [56,73]. This loss of pericyte coverage constitutes the earliest structural lesion of vasodegeneration, destabilizing the microvasculature and predisposing the endothelial layer to structural breakdown.

The failure to resolve these cell-specific quality control defects culminates in sustained inflammation and long-term pathology. The leakage of damaged mtDNA into the cytoplasm acts as a potent DAMP, robustly activating the innate immune cGAS-STING signaling pathway [90]. This DR-specific inflammatory axis serves as a central hub, initiating NF-κB and Type I interferon responses that drive the sustained production of permeability enhancers like VEGF and IL-6, which directly cause the degradation of tight junction proteins ZO-1, Occludin, Claudin-5 [91,92]. Furthermore, the long-term impact of hyperglycemia is encoded through metabolic memory, where chronic mitochondrial stress—such as persistent Drp1 activation or *MFN2* promoter methylation—induces stable epigenetic modifications. These modifications lock the retinal cells into a vulnerable state, perpetuating iBRB dysfunction even after subsequent glycemic control [93]. Collectively, DR pathology represents a complex interplay of HBP/O-GlcNAcylation-driven stress, a cell-specific quality control paradox, and mtDNA-mediated sterile inflammation, all converging to synergistically dismantle the inner blood-retinal barrier (Table 2). Future therapeutic strategies must therefore aim to restore mitochondrial plasticity and temper inflammatory amplification in a mechanism- and cell-type-specific manner [94,95].

### 3.2. Retinal Vein Occlusion (RVO)

RVO is a major cause of vision loss characterized by venous obstruction, ischemia–reperfusion (I/R) injury, and secondary breakdown of the iBRB. Unlike the chronic metabolic stress observed in DR, RVO manifests as an acute-onset vascular occlusive event that induces secondary I/R fluctuations, imposing abrupt energetic and oxidative stress on retinal microvascular cells. This distinct pathogenic environment provokes a unique, biphasic mitochondrial response—an initial phase of catastrophic energy collapse followed by a reperfusion phase marked by oxidative and inflammatory amplification.

During ischemia, impaired OXPHOS rapidly depletes ATP and drives a switch to anaerobic glycolysis, leading to cytosolic acidosis and accumulation of metabolic intermediates. The abrupt restoration of oxygen during reperfusion then accelerates electron leakage from complexes I and III of the ETC, resulting in a burst of mitochondrial mtROS. This “oxidative surge” destabilizes mitochondrial membranes, promotes the opening of the mPTP, and triggers massive calcium influx, culminating in mitochondrial swelling and endothelial apoptosis. Importantly, this injury pattern is transient and spatially heterogeneous—central ischemic regions undergo necrosis, whereas peripheral zones display reversible mitochondrial depolarization, reflecting an opportunity for therapeutic rescue.

Beyond oxidative damage, reperfusion-induced mitochondrial stress activates sterile inflammatory pathways that perpetuate vascular injury. Leakage of mitochondrial components, including mtDNA and N-formyl peptides, engages pattern recognition receptors such as TLR9 and cGAS–STING, initiating NF-κB and type I interferon cascades that upregulate IL-6, TNF-α, and VEGF [36,96,97,98]. These mediators increase endothelial permeability and promote leukocyte adhesion, exacerbating macular edema [99]. Unlike chronic inflammatory priming in DR, this inflammation in RVO is episodic and time-dependent—transient activation may contribute to tissue repair and angiogenesis, whereas prolonged activation leads to irreversible barrier breakdown [100].

Mitochondrial dynamics in RVO follow a temporal dual-phase pattern. During acute ischemia, excessive activation of Drp1 drives mitochondrial fragmentation and cytochrome c release, while fusion mediators MFN2 and OPA1 are downregulated [101,102]. However, during the early reperfusion phase, controlled fission is essential for removing damaged mitochondria through PINK1/Parkin-dependent mitophagy and for metabolic recovery. Prolonged or uncontrolled Drp1 activity, conversely, prevents mitochondrial network restoration and sustains endothelial cell death. These observations indicate that the therapeutic goal in RVO is not persistent Drp1 inhibition but temporally optimized modulation of mitochondrial fission–fusion balance.

Pericyte mitochondrial dysfunction also plays a decisive role in RVO-induced iBRB instability. ATP depletion and calcium overload impair pericyte contractility, disrupting microvascular tone and endothelial support [103]. In addition, pericyte apoptosis reduces mural coverage, amplifying vascular leakage. Crosstalk between stressed endothelial and pericyte mitochondria—via ROS, cytokines, and gap junction signals—creates a feed-forward loop that accelerates barrier collapse and tissue hypoxia [7,104,105,106].

Emerging evidence suggests that targeted restoration of mitochondrial homeostasis offers a promising therapeutic avenue. Preclinical studies demonstrate that transient Drp1 inhibition, enhancement of PINK1-dependent mitophagy, or blockade of mtDNA-induced inflammation can attenuate oxidative injury, reduce vascular leakage, and preserve barrier integrity [100,107]. Future research should emphasize spatiotemporal precision therapy—using mitochondria-targeted nanoparticles or real-time metabolic imaging—to monitor and restore mitochondrial function dynamically during the ischemia–reperfusion cycle [108] (Table 3). Achieving this level of control could transform RVO management from symptomatic treatment to true mitochondria-centered vascular protection. 

## 4. Therapeutic Strategies

**Figure 3:**
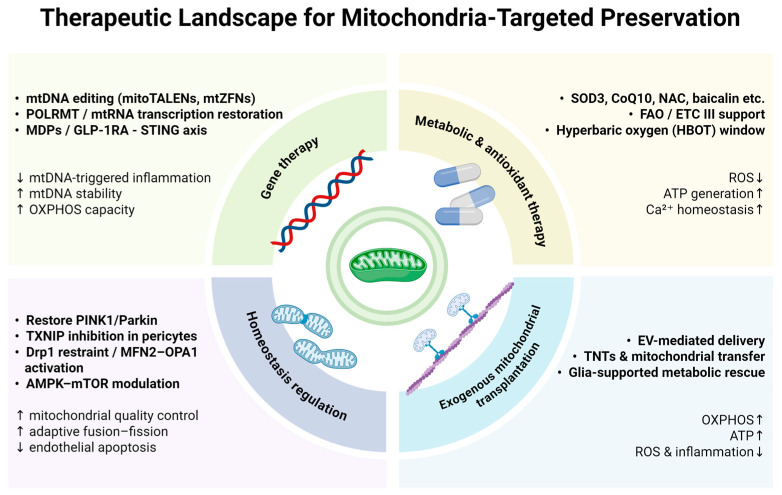
Landscape of mitochondria-targeted therapeutic strategies for preserving iBRB integrity. This figure summarizes four major therapeutic categories—gene therapy, metabolic and antioxidant modulation, mitochondrial homeostasis regulation, and exogenous mitochondrial transplantation—and illustrates how these strategies restore OXPHOS capacity, stabilize redox balance, and reinforce the structural resilience of the iBRB, thereby collectively supporting barrier preservation. The arrows indicate the convergent mitochondrial consequences of these interventions, including reduced mtDNA-triggered inflammation and enhanced mtDNA stability, improved ROS handling and Ca^2+^ homeostasis, recovery of OXPHOS efficiency and ATP generation, and attenuation of endothelial apoptosis and inflammatory signaling, thereby supporting the maintenance of iBRB integrity. The figure was created in BioRender (Chen, Z. (2025) https://BioRender.com/60sjo8t).

Mitochondrial dysfunction has emerged as both a hallmark and a driver of iBRB breakdown, positioning mitochondria at the crossroads of retinal vascular degeneration and neuroinflammation. Consequently, therapeutic strategies have shifted from symptom alleviation toward the precise restoration of mitochondrial integrity and signaling. A growing body of research supports a multidimensional approach encompassing gene therapy, metabolic modulation, homeostatic regulation, and mitochondrial transplantation. Gene-based interventions aim to correct or compensate for mtDNA defects and restore bioenergetic competence, while pharmacologic strategies targeting mitochondrial metabolism and oxidative stress seek to reestablish redox equilibrium and sustain cellular vitality. Beyond these functional restorations, regulating mitochondrial quality control—including the orchestration of mitophagy, fusion–fission dynamics, and protein import—has shown promise in rebalancing energy turnover and preventing irreversible barrier collapse. At the frontier of innovation, exogenous mitochondrial transplantation introduces the prospect of direct organelle replacement and intercellular metabolic coupling within the iBRB microenvironment. Collectively, these mitochondria-targeted approaches represent a paradigm shift—from treating retinal vascular damage as a downstream manifestation to addressing mitochondrial failure as the origin of disease progression (Figure 3).

### 4.1. Gene Therapy

Gene therapy strategies aim to correct or compensate for mitochondrial genomic defects. Direct mtDNA editing, utilizing engineered nucleases like mitoTALENs and mtZFNs, offers a precise method to selectively degrade mutated mtDNA and restore genomic integrity [109]. However, this approach confronts significant translational hurdles, including the efficiency of mitochondrial delivery, the risk of off-target cleavage, and the long-term stability of edited genomes in post-mitotic tissues such as the retina.

Beyond direct editing, broader gene-based strategies focus on functional compensation. The delivery or overexpression of key mitochondrial enzymes and transcriptional regulators can reverse functional deficits, enhance bioenergetic capacity, and improve cellular survival [29,30]. Repairing defective mtDNA, for example, has shown promise in improving retinal function and slowing DR progression [110].

Emerging concepts also include modulating pathways that influence mitochondrial gene expression. Mitochondrial-derived peptides (MDPs), encoded by short open reading frames (sORFs) within mtDNA, are known regulators of metabolism, aging, and inflammation [111]. Similarly, GLP-1 receptor agonists (GLP-1 RAs) may enhance mitochondrial gene translation and function, potentially offering a multi-tiered intervention by suppressing STING pathway activation [23]. However, the precise roles of MDPs and GLP-1 RAs in human iBRB regulation remain speculative, with most evidence derived from systemic metabolic models.

### 4.2. Energy Metabolism Regulation and Antioxidant Therapy

Targeting mitochondrial energy metabolism and oxidative stress constitute core strategies to protect the iBRB. Enhancing respiratory chain activity to restore mitochondrial metabolism, such as maintaining complex III function or promoting fatty acid oxidation, can promote endothelial functional recovery and maintain vascular homeostasis [27,61].

Antioxidant therapies, such as SOD3, not only suppress excessive ROS generation but also regulate calcium homeostasis, thereby improving the metabolic state and barrier function [42,66,112,113,114]. It is noteworthy that the effects of antioxidants exhibit significant context dependence—while short-term ROS scavenging aids in restoring redox balance, excessive antioxidant exposure may impair physiological signaling and suppress mitochondrial adaptive responses. Natural compounds (e.g., baicalin derivatives) [115,116,117] and classical antioxidants (NAC, coenzyme Q10, resveratrol, and melatonin) [118,119,120,121] have been shown to reduce ROS accumulation, restore mitochondrial membrane potential, and attenuate apoptosis. However, differences in bioavailability, dose sensitivity, and efficacy evaluation criteria significantly limit their clinical translation. In addition, long-term hyperbaric oxygen therapy (HBOT) has been reported to improve mitochondrial function and mitigate oxidative stress. But the hyperoxic environment may activate mitochondria while also increasing oxidative stress, resulting in a narrow therapeutic window for HBOT [122].

Meanwhile, metabolic coupling between pericytes and endothelial cells is also essential for barrier stability; high-glucose-induced pericyte fragmentation or tunneling nanotube (TNT)–mediated metabolic support can significantly influence endothelial resilience and iBRB integrity [50,51].

### 4.3. Regulation of Mitochondrial Homeostasis

Correcting mitochondrial homeostasis—particularly autophagic quality control—offers cell-type—tailored opportunities. In endothelial cells, pro-mitophagy interventions that restore PINK1/Parkin signaling or receptor-mediated clearance (BNIP3/FUNDC1) lower mtROS, preserve tight junctions, and reduce apoptosis [36,123]. Rebalancing AMPK–mTORC1 re-establishes autophagic flux and dampens cytokine release [124,125,126,127]. The PI3K-AKT-mTORC1 axis interacts with mitochondrial quality control. Inhibiting any component of this pathway significantly reduces mutational burden, restores mitochondrial bioenergetic function, and decreases glucose dependence, suggesting this signaling pathway may serve as a potential therapeutic target. However, how these pathways selectively clear mutated mtDNA remains unclear and warrants mechanistic dissection [128]. By contrast, pericytes often display over-clearance: TXNIP-driven, Drp1-biased mitophagy correlates with mural-cell loss and vasodegeneration; tempering TXNIP signaling or downstream clearance preserves pericyte coverage and capillary stability [129]. Where fragmentation biases mitochondria toward removal, moderate Drp1 restraint curbs apoptosis and improves contractile function [103,130].

Regulating mitochondrial fusion and fission is also crucial [131]. Under hyperglycemia/oxidative stress, Drp1/Fis1-dominant fission drives fragmentation, cytochrome-c release, and barrier leak; pharmacologic or genetic Drp1 restraint reduces permeability and improves endothelial migration/repair [48,130]. Conversely, supporting fusion proteins MFN2/OPA1 restores network competence and respiration, stabilizing barrier proteins [132,133]. Yet these regulatory approaches still face inherent challenges in translational application. Fusion/fission regulators are widely distributed across multiple tissues, and systemic interventions may induce off-target mitochondrial remodeling beyond the retina. Adjacent to dynamics, protein-import homeostasis (e.g., TIMM44) emerges as a tractable lever: enhancing mitochondrial import sustains angiogenic capacity and endothelial viability [134]. Targeting TRAP1 to modulate mitochondrial proteostasis can promote HIF-1α degradation, lower VEGF, and ameliorate ischemic retinopathy [135,136]. A significant limitation across this field is that most current research remains focused on the sustained effects of “acute functional improvement” rather than “structural repair.” Therefore, the application of homeostasis modulators requires precise temporal, spatial, and cellular-level control.

### 4.4. Exogenous Mitochondrial Transplantation

**Figure 4:**
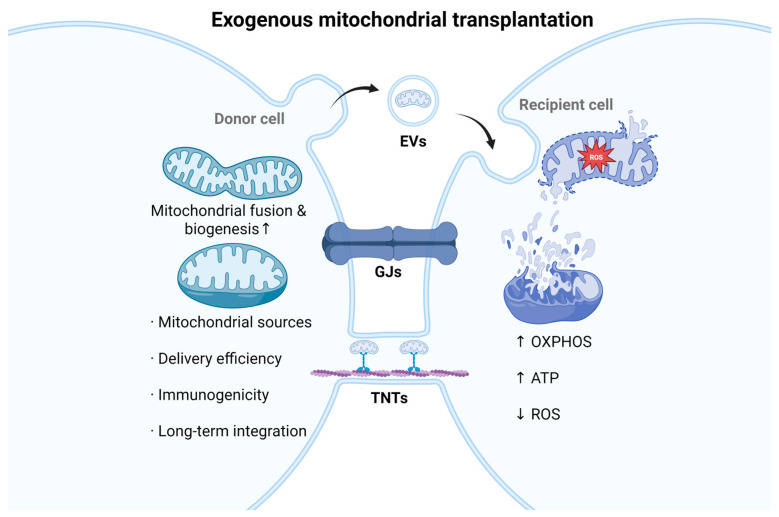
Mechanisms of mitochondrial transfer and exogenous mitochondrial transplantation (MTx). Exogenous mitochondrial transplantation (MTx) involves the delivery of bioenergetically competent mitochondria into injured cells or tissues to restore mitochondrial integrity and metabolic homeostasis. Intercellular mitochondrial transfer can be mediated through tunneling nanotubes (TNTs), extracellular vesicles (EVs), and gap junctions (GJs). These communication routes enable the exchange of functional mitochondria and facilitate the removal of damaged ones, collectively enhancing oxidative phosphorylation (OXPHOS), restoring ATP synthesis, and reducing ROS accumulation, as indicated by the arrows. By improving redox balance and bioenergetic capacity, mitochondrial transplantation holds therapeutic potential for preserving iBRB integrity under mitochondrial stress. The abbreviations in the figure are defined as follows: TNTs, tunneling nanotubes; EVs, extracellular vesicles; and GJs, gap junctions. It was created in BioRender (Chen, Z. (2025). https://BioRender.com/9ulegpw).

Exogenous mitochondrial transplantation (MTx) is an emerging therapy that introduces healthy mitochondria into damaged cells or tissues to restore mitochondrial function and cellular vitality [137]. Mitochondria can be transferred between cells through several intercellular communication routes, including TNTs, extracellular vesicles (EVs), and gap junctions (GJs) (Figure 4). Under conditions of energetic stress, inflammatory stimulation, or DNA damage, elevated intracellular ROS levels activate these transfer mechanisms [138].

Preclinical studies are encouraging. Transplantation of healthy mitochondria into high-glucose–injured endothelial cells improved respiration and preserved tight junction proteins, thereby maintaining iBRB integrity [139]. Delivery of mitochondria via extracellular vesicles to damaged iBRB regions can restore OXPHOS capacity, enhance ATP generation, reduce ROS, and improve redox balance [140,141]. Furthermore, mitochondrial supplementation in Müller glia and astrocytes can restore glutamate metabolism, indirectly protecting barrier function from excitotoxicity [53,142].

Although these findings are encouraging, mitochondrial transfer itself remains a double-edged sword. While it can enhance energy metabolism and alleviate oxidative stress, under chronic inflammatory conditions, it may also promote the intercellular spread of damaged or heterogeneous mitochondria, leading to secondary damage. The detailed mechanisms and regulatory factors governing mitochondrial exchange remain to be fully elucidated. Although intercellular mitochondrial transfer has been observed in vitro, its occurrence and significance in vivo have yet to be confirmed. Exogenous MTx similarly faces significant translational challenges, including standardization of mitochondrial sources, delivery efficiency, immunogenicity, and long-term integration with host cellular networks. Overcoming these challenges will require spatiotemporally controlled delivery systems and real-time in vivo tracking technologies to enable safe, precise, and durable clinical translation.

## 5. Summary

Mitochondria stand at the crossroads of metabolism, signaling, and survival within the iBRB. Far beyond their classical role as bioenergetic engines, they integrate oxidative flux, calcium homeostasis, and organelle quality control into a unified system that sustains vascular integrity. When this coordination fails—through mtDNA instability, redox imbalance, or disrupted fusion–fission dynamics—localized metabolic stress propagates into systemic vascular collapse. Across retinal pathologies, this breakdown takes divergent temporal forms: DR imposes a slow, epigenetically imprinted metabolic drift, whereas retinal vein occlusion unleashes an acute ischemia–reperfusion storm. Yet both trajectories converge on a single mechanistic truth—the erosion of mitochondrial adaptability.

This realization redefines mitochondrial dysfunction as the central rheostat of iBRB fate. It implies that effective therapy should not pursue linear correction of single targets, but rather the restoration of mitochondrial plasticity—the capacity to recalibrate energy production, redox balance, and organelle turnover in real time. Emerging approaches, from mitophagy reactivation and fusion–fission tuning to mitochondrial gene modulation and organelle transplantation, illustrate how precise manipulation of mitochondrial signaling can reverse vascular decline. Yet these interventions remain constrained by a lack of temporal control, incomplete understanding of cell-type specificity, and insufficient tools for real-time mitochondrial imaging in vivo.

The next phase of research must therefore move toward spatiotemporally resolved, mechanism-guided therapeutics, integrating mitochondrial-targeted nanocarriers, spatial multi-omics, and metabolic imaging to capture the dynamic interplay between mitochondrial state and vascular behavior. Conceptually, this represents a shift from viewing mitochondria as passive victims of retinal disease to recognizing them as active arbiters of vascular fate. Mastering their regulation may ultimately allow us to convert mitochondrial vulnerability into resilience—transforming the iBRB from a fragile interface into a metabolically intelligent barrier capable of self-repair and long-term stability. Bridging mechanistic understanding with translational precision will define the next era of mitochondria-centered retinal therapy.

## Figures and Tables

**Table 1:** Mechanisms by which mitochondrial dysfunction contributes to cellular damage within the inner blood-retinal barrier (iBRB).

Cell Type	Mechanism	Effects on the iBRB	References
Endothelial cells	mtDNA mutations and reduced copy number impair OXPHOS	Energy depletion and oxidative stress trigger endothelial apoptosis, inflammatory activation, and tight-junction disruption	[13,14,15,16,17,18,19,20,21,22,23,24,25]
	Impaired ATP synthesis and dysfunction of mitochondrial ETC complexes I/III	Loss of cytoskeletal stability and tight-junction integrity; increased endothelial permeability	[26,27,28,29,30,31,32,33,34]
	Excessive ROS generation via ETC complexes I/III and activation of NF-κB/MAPK signaling	Amplified oxidative stress, endothelial apoptosis, degradation of junctional proteins (ZO-1, Occludin), and up-regulation of pro-inflammatory mediators	[35,36,37,38]
	Defective PINK1/Parkin-mediated mitophagy and Fundc1-dependent flux blockade	Accumulation of damaged mitochondria leads to chronic ROS elevation and endothelial cell death	[39,40,41]
	Disturbed Ca^2+^ homeostasis via TRPV4–MCU overactivation and VDAC1–GRP75–IP3R coupling	Mitochondrial Ca^2+^ overload, mPTP opening, ROS bursts, cytoskeletal collapse, and barrier leakage	[42,43,44,45,46]
	Imbalance between mitochondrial fusion and fission (Drp1/Fis1, MFN2/OPA1)	Mitochondrial fragmentation, cytochrome c release, reduced stress adaptability, and endothelial barrier disruption	[47,48]
Pericytes	Mitochondrial fragmentation and metabolic impairment	Decreased contractility, microvascular destabilization, and impaired support for endothelial cells	[30,49,50,51,52]
	TXNIP-mediated excessive mitophagy and oxidative stress	Mitochondrial depletion, apoptosis, and pericyte loss leading to capillary regression	[39,40]
Glial cells	ER–mitochondrial crosstalk (PERK/eIF2α/JAK1 axis) and redox imbalance	Enhanced cytokine release, loss of neurovascular coupling, and diminished barrier-supportive functions	[53,54,55,56] ^1^

^1^ This table summarizes the principal mechanisms by which mitochondrial dysfunction contributes to cellular injury within the iBRB. Alterations in mtDNA stability, bioenergetics, ROS generation, Ca^2+^ homeostasis, mitophagy, and mitochondrial dynamics interact across endothelial, pericyte, and glial compartments, culminating in barrier breakdown. OXPHOS, oxidative phosphorylation; ETC, electron transport chain; ROS, reactive oxygen species; PINK1, PTEN-induced kinase 1; FUNDC1, FUN14 domain-containing protein 1; TRPV4, transient receptor potential vanilloid 4; MCU, mitochondrial calcium uniporter; VDAC1, voltage-dependent anion channel 1; GRP75, glucose-regulated protein 75; IP3R, inositol 1,4,5-triphosphate receptor; mPTP, mitochondrial permeability transition pore; Drp1, dynamin-related protein 1; MFN2, mitofusin 2; OPA1, optic atrophy 1; TXNIP, thioredoxin-interacting protein; PERK, protein kinase RNA-like ER kinase; JAK1, Janus kinase 1.

**Table 2:** Key mechanisms of mitochondrial dysfunction in diabetic retinopathy (DR).

Mechanism	Key Pathways	DR-Specific Alterations and Effects on iBRB Cells	References
mtDNA mutations and instability	Reduced mtDNA copy number; *ND1*/*ND6*; cytochrome b; *POLRMT* deficiency; *AKAP1* loss	High glucose increases mtDNA damage and cytoplasmic mtDNA leakage, activating cGAS–STING and NF-κB, which trigger VEGF/IL-6 release and endothelial inflammation	[90,91,92]
Energy deficiency and OXPHOS impairment	Complex I/III dysfunction; *NDUFS8* loss; *POLRMT* depletion; ATP synthase inhibition	Persistent ATP shortage reduces endothelial migration and barrier repair, weakens pericyte contractility, and impairs neurovascular coupling under hyperglycemia	[82,83]
ROS generation and oxidative stress	Complex I/III—derived ROS; NOX activation via AGEs–RAGE; NF-κB/MAPK pathways	Hyperglycemia and AGEs drive excessive ROS, causing endothelial apoptosis, tight-junction degradation (ZO-1, Occludin), and pericyte cytoskeletal oxidation	[82,83,84]
Mitophagy dysregulation	Endothelium: PINK1/Parkin deficiency and mTOR overactivation; Pericytes: TXNIP-driven excess mitophagy	Endothelial cells retain damaged mitochondria → mtROS and apoptosis; Pericytes undergo mitochondrial depletion → bioenergetic collapse and cell loss	[85,86,87,88,89]
Fusion–fission imbalance	Drp1/Fis1 upregulation; MFN2/OPA1 downregulation; PGC-1α suppression	O-GlcNAc-modified Drp1 activation and loss of fusion proteins promote mitochondrial fragmentation and apoptosis, driving progressive iBRB breakdown	[82,83,85,86,87,88,89]
Epigenetic metabolic memory	Persistent Drp1 activation; *MFN2* promoter methylation; histone modifications	Long-term mitochondrial stress induces stable epigenetic reprogramming, maintaining oxidative and inflammatory vulnerability after glycemic normalization	[93] ^1^

^1^ Each pathway highlights distinct yet interconnected alterations in mitochondrial integrity, energy metabolism, and redox regulation within iBRB structural cells. These maladaptive responses collectively drive endothelial inflammation, pericyte loss, and barrier breakdown under hyperglycemia. AGEs, advanced glycation end-products.

**Table 3:** Key mechanisms of mitochondrial dysfunction in retinal vein occlusion (RVO).

Mechanism	Key Pathways	RVO-Specific Alterations and Effects on iBRB Cells	References
Acute energy deficit	Venous obstruction; acute ischemia; OXPHOS inhibition	Rapid ATP depletion, shift to anaerobic glycolysis, cytosolic acidosis, and early endothelial energy failure that destabilizes the barrier	[83,84,103]
Reperfusion-driven oxidative burst	Electron leakage from ETC complexes I/III; mPTP opening; Ca^2+^ influx	Intense oxidative surge leading to mitochondrial swelling and endothelial apoptosis, with transient and spatially heterogeneous injury patterns characterized by central necrosis and peripheral reversible depolarization	[96,100,101,102]
mtDNA-mediated sterile inflammation	Leakage of mtDNA/N-formyl peptides; activation of TLR9 and cGAS–STING;NF-κB/type I IFN	Upregulation of IL-6, TNF-α, and VEGF, increased endothelial permeability, and enhanced leukocyte adhesion; inflammation exhibits time-dependent behavior, where transient activation may aid repair but sustained activation drives irreversible breakdown	[36,96,97,98,99]
Biphasic changes in mitochondrial dynamics	Ischemia: excessive Drp1 activation; MFN2/OPA1 downregulation Reperfusion: PINK1/Parkin-dependent clearance	During ischemia, excessive mitochondrial fragmentation and cytochrome-c release occur, while the early reperfusion phase requires controlled fission to remove damaged mitochondria; persistent Drp1 overactivation impairs network recovery and prolongs endothelial loss	[100,101,102]
Pericyte mitochondrial dysfunction	ATP depletion; Ca^2+^ overload; ROS elevation	Impaired pericyte contractility and apoptosis reduce mural coverage, weaken microvascular support, and exacerbate vascular leakage	[7,103,106]
Endothelial–pericyte mitochondrial crosstalk	ROS–cytokine feedback; gap-junction signaling	Bidirectional amplification of oxidative and inflammatory signals generates a feed-forward injury loop that promotes sustained iBRB instability and worsening hypoxia	[7,104,105,106] ^1^

^1^ Each pathway reflects the acute and temporally distinct mitochondrial disturbances triggered by venous obstruction and ischemia–reperfusion injury in RVO. These alterations converge on impaired bioenergetics, excessive oxidative signaling, dysregulated mitochondrial dynamics, and sterile inflammation within iBRB structural cells. Together, these maladaptive responses exacerbate endothelial instability, pericyte loss, microvascular leakage, and progressive barrier breakdown under ischemic stress. mPTP, mitochondrial permeability transition pore; IFN, interferon; ETC, electron transport chain.

## Data Availability

No new data were created or analyzed in this study. Data sharing is not applicable to this article.
